# The functional and predictive roles of miR-210 in cryptorchidism

**DOI:** 10.1038/srep32265

**Published:** 2016-08-26

**Authors:** Zhengzheng Duan, Helong Huang, Fei Sun

**Affiliations:** 1 Department of Cell and Developmental Biology, School of Life Sciences, University of Science and Technology of China, Hefei, Anhui 230027, China; 2 Hefei National Laboratory for Physical Sciences at Microscale and School of Life Sciences, University of Science and Technology of China, Hefei, Anhui 230026, China

## Abstract

Idiopathic diseases of the reproductive system are important factors leading to male infertility. Many studies have shown that microRNAs (miRNAs) regulate the expression of multiple genes that play a significant role in spermatogenesis and development. We previously showed that microRNA-210 (miR-210) is one of the markedly upregulated microRNAs in the testes of sterile males with maturation arrest (MA). However, the role of miR-210 in spermatogenesis remains unknown. In this study, we found that miR-210 is highly expressed not only in patients with MA but also in patients with cryptorchidism. In addition, miR-210 inhibits the expression of Nuclear Receptor Subfamily 1, Group D, Member 2 (NR1D2) both *in vitro* and *in vivo*, particularly in cryptorchidic tissues. To facilitate further research, we established a mouse model of cryptorchidism and were surprised to discover that the miR-210 expression pattern was in accordance with that of patients with cryptorchidism. Thus, we propose that miR-210 may serve as a biomarker of cryptorchidism in clinical tests.

Infertility occurs at a rate of 10–15% among couples of childbearing age, and half of these cases are thought to be due to male infertility^1^. Furthermore, patients with unexplained non-obstructive azoospermia (NOA), particularly men with maturation arrest (MA, a cause of NOA), usually exhibit a significantly lower sperm retrieval rate and their sperm leads to a lower clinical pregnancy rate. In addition to a variety of external factors, some common conditions, such as cryptorchidism (also known as undescended testes) and varicocele, affect spermatogenesis and are significant causes of male infertility^2^,^3^,^4^.

During normal development, a testis descends from the waist to the scrotum via the retroperitoneal space. When this migration fails or remains incomplete, the scrotum will have no or at most one testis. This phenomenon is called cryptorchidism or undescended testis^4^,^5^,^6^. Cryptorchidism is a common congenital disease of the genitourinary system that negatively affects male infants. The incidence of cryptorchidism is approximately 2 to 4%; however, 30% of premature male infants are affected^4^. Although there are several hypotheses to explain cryptorchidism, the underlying pathological basis is not yet known. Cryptorchidism has profound influences, particularly on normal spermatogenesis and fertility potential. Additionally, one of the current perspectives holds that cryptorchidism influences testicular function and increases the risk of testicular cancer^7^,^8^.

Recently, many studies have suggested that microRNAs (miRNAs) play an important role in spermatogenesis, and their dysregulation has been implicated in various diseases that lead to infertility^9^,^10^,^11^. MicroRNAs are short noncoding RNA molecules (typically 19–23 nucleotides in length) that regulate the post-transcriptional and translational efficiency of target mRNAs through base pairing with their 3′-untranslated regions (3′-UTRs)^12^.

Our previous analyses of microRNA arrays showed that microRNA-210 (miR-210) expression is upregulated in the testes of infertile men with maturation arrest (MA)^13^. miR-210 is an established target of hypoxia-inducible factors, and its upregulation is a characteristic of hypoxic conditions. Because hypoxia is an essential characteristic in the neoplastic microenvironment, the consistent upregulation of miR-210 under hypoxic conditions is a signature characteristic of solid tumours^14^. Currently, most investigations of miR-210 function have focused on its relationship with various types of cancer, including pancreatic cancer^15^ and lung cancer^16^,^17^. In addition, miR-210 expression has been evaluated as a prognostic factor in breast cancer^18^.

Clinical research involving miR-210 has focused on cancer diagnoses; thus, its expression and function in the reproductive system remains unknown. Here, we characterized the expression of miR-210 in the reproduction system and demonstrated that NR1D2 (Nuclear Receptor Subfamily 1, Group D, Member 2) is a specific target gene of miR-210. To investigate the potential role of miR-210 in spermatogenesis and cryptorchidism, we studied the correlation between miR-210 and NR1D2 in the testicular embryonic carcinoma cell line NTERA-2 (NT-2) and explored potential targets of miR-210 that may participate in testis development.

## Materials and Methods

### Tissue samples

Human testicular tissue samples from control individuals and patients with MA were obtained from the First Affiliated Hospital of Anhui Medical University (Hefei, China). All patients signed informed consent documents approving the use of their tissues for research purposes. Written informed consent, which conformed to the tenets of the Declaration of Helsinki, was obtained from each participant prior to the study. This study received ethical approval from the institutional review boards of the University of Science and Technology of China and Anhui Medical University. All of the methods strictly abided by the ethical review organizations’ guidelines.

### Animals

Mice were purchased from the Shanghai Laboratory Animal Center (SLAC) and maintained in a specific pathogen-free (SPF) animal facility. The mice were kept at 22 °C with a 14 h light/10 h dark light cycle; they were provided food and water ad libitum. The surgeries were performed while mice were narcotized. Testicular tissues were obtained after the mice were sacrificed by cervical dislocation. All mouse experiments were performed in accordance with the relevant guidelines and regulations. This study received ethical approval from the institutional review board of the University of Science and Technology of China.

### Cell culture and transfection microRNAs, siRNAs and plasmids

NTera-2 (NT-2) cells are derived from human embryonic carcinomas. Cells (NT-2 and HEK293T) were cultured in Dulbecco’s modified Eagle’s medium (DMEM), supplemented with 1% antibiotics (100 U/ml penicillin and 100 mg/ml streptomycin, Life Technologies Inc., Grand Island, NY, USA) and 10% (v/v) foetal bovine serum (Life Technologies Inc.). The cells were cultured at 37 °C in a humidified incubator with 5% carbon dioxide. We used Lipofectamine RNAiMAX (Invitrogen, Carlsbad, CA, USA) and X-tremeGENE HP DNA Transfection Reagent (Roche) to transfect NT-2 cells with oligonucleotides and plasmids. Lipofectamine 2000 Reagent (Invitrogen) was used to transfect 293T cells. All transfection procedures were performed following the manufacturer’s instructions.

Shanghai Gene-Pharma Co. (Shanghai, China) synthesized and optimized duplex miR-210 mimics and a miR-210 inhibitor as well as a negative control (NC) and an NC inhibitor. The inhibitor of miR-210 is single-stranded, sequence-specific, and chemically modified to specifically target and knock down miR-210 molecules. NR1D2 siRNA (si.NR1D2) was also designed and synthesized by Shanghai Gene-Pharma Co. The si.NR1D2 sequences were as follows: sense, 5′-GCAUGGUUCUGUGUAATT-3′; antisense, 5′-UUACACAGAACCAUGCTT-3′.

The psiCHECK-2 dual-luciferase reporter plasmid was provided by Biliang Zhang (Guangzhou Institute of Biomedicine and Health, Chinese Academy of Sciences, China), and the p3XFLAG-myc-CMV™-24 expression vector was purchased from Sigma. The pEGFP-C1 vector was purchased from BD Biosciences Clontech. Wild-type (WT) plasmids of the NR1D2 3′-UTR were constructed by amplifying a 580-bp 3′-UTR fragment of NR1D2 mRNA and 2258-bp CDS plus 3′-UTR fragment of NR1D2 mRNA harbouring the miR-210 binding site predicted by miRanda (http://www.microrna.org) and DNAman, whereas mutated (MT) NR1D2 3′-UTR was generated by PCR-based site-directed mutagenesis. The WT and MT NR1D2 3′-UTR fragments were fused with the psiCHECK-2 reporter vector at the NotI and XhoI sites. The primers were as follows:

WT NR1D2 3′-UTR

Forward primer: 5′-TGCCTCGAGCTTCAGATGATTAGACGT-3′

Reverse primer: 5′-ATTGCGGCCGCCATATGGCAGGAACCCTGAA-3′

MT NR1D2 3′-UTR

Forward primer: 5′-CAATATAACCGTCAATCACAAG -3′

Reverse primer: 5′-CTTGTGATTGACGGTTATATTG -3′

WT NR1D2 CDS plus 3′-UTR in p3XFLAG vector

Forward primer: 5′-ATTGAATTCTATGAAAACAAGCAAATCGAG -3′

Reverse primer: 5′-TGCGTCGACCATATGGCAGGAACCCTGAA-3′

WT NR1D2 CDS plus 3′-UTR in pEGFP vector

Forward primer: 5′-ATTGAATTCTATGAAAACAAGCAAATCGAG -3′

Reverse primer: 5′-TGCGTCGACTTAAGGGTGAACTTTAAAGGC -3′

### Luciferase reporter assay

Luciferase activity was detected using the Dual Luciferase Reporter Assay System (Promega Biotech Co., Ltd, Beijing) after incubating transfected cells for 30 h to allow for the expression of transfected DNA. Renilla luciferase activity was normalized to the firefly luciferase activity in each well. All experiments were repeated in triplicate. Either 40 nM of miR-210 mimic or miR-210 inhibitor were co-transfected with 200 ng of psiCHECK-2 vectors into 293T cells in 24-well plates.

### Western blotting

Cells were lysed in RIPA buffer (50 mM Tris-HCl, pH 7.4, 150 mM, NaCl, 1% Triton X-100, 1% sodium dodecyl sulfate, 1% sodium deoxycholate, and 1 mM EDTA) containing a completely EDTA-free protease inhibitor cocktail (Roche), 1 mM phenylmethylsulfonyl fluoride (PMSF) and phosphatase inhibitors (5 mM sodium orthovanadate). Protein lysates were loaded on SDS-PAGE gels and electroblotted onto nitrocellulose membranes (Amersham Biosciences). The nitrocellulose membranes were blocked for 1 h in 5% non-fat milk in TBST (10 mM Tris, pH 7.5, 200 mM NaCl, and 0.2% Tween 20), followed by incubation with primary antibodies. The antibodies used for Western blotting analysis were anti-NR1D2 (Proteintech Group, Inc.) and anti-Actin (Abcam, Cambridge, MA, USA).

### *In situ* hybridization

ISH was performed on 10-mm frozen tissue sections using LNA-modified DNA probes. The probe sequences are listed in Supplementary Table 2. Briefly, 10-mm testis biopsy sections obtained from normal controls and NOA patients were fixed with 4% paraformaldehyde for 15 min at room temperature. To block endogenous alkaline phosphatase activity, the slides were immersed and stirred gently in 0.1 M ethanolamine and 2.5% acetic anhydride for 10 min, followed by treatment with 5 mg/ml proteinase K for 3 min after extensive washing with PBS. Prehybridizations were performed for 6 h in a hybridization oven between 21 and 23 °C, which is below the reported melting temperature of the LNAs (57 °C), with 700 ml of prehybridization buffer [50% formamide, 5 × SSC, 5 × Denhardt’s, 200 mg/ml yeast RNA, 500 mg/ml salmon sperm DNA, 2% Roche blocking reagents (Roche, Basel, Switzerland) and DEPC-treated water]. A probe (1 pmol) was added to 150 ml of denaturing hybridization buffer (50% formamide, 5 × SSC, 5 × Denhardt’s, 200 mg/ml yeast RNA, 500 mg/ml salmon sperm DNA, 2% Roche blocking reagents, 0.25% CHAPS, 0.1% Tween and DEPC-treated water). After denaturing at 80 °C for 5 min, hybridization occurred overnight on the prehybridization temperature-covered glass coverslips. To remove the coverslips, the slides were soaked in pre-warmed 60 °C 5 × SSC. After incubation in 0.2 × SSC at 60 °C for 1 h, the sections were washed in B1 solution (0.1 M Tris pH 7.5/0.15 M NaCl) at room temperature for 10 min. After blocking for 1 h in 20% sheep serum (Santa Cruz Biotechnology Inc.) diluted with B1 solution, the sections were incubated overnight at 4 °C in 10% sheep serum containing anti-Digoxigenin-AP FAB fragments (Roche; 1:250). After washing three times for 5 min each in the B1 solution at room temperature and equilibrating for 10 min in B3 solution (0.1 M Tris pH 9.5/0.1 M NaCl/50 mM MgCl_2_), the sections were stained with NBT/BCIP (Roche) overnight at room temperature. When each probe yielded a strong signal or the NCs began to show background signal, the reactions were stopped by washing with PBS. The signals were visualized by standard light microscopy.

### Histological analysis and immunohistochemistry (IHC)

IHC was conducted to localize the NR1D2 protein in human testicular tissues. Human testes were dissected into pieces, fixed with 4% PFA, embedded in paraffin, and sectioned at 4 um. To confirm the specific infertility syndrome, sections were stained with haematoxylin and eosin following a standard protocol. Initially, slides with testicular tissue sections were heated in 10 mM sodium citrate buffer (pH 6.0) for 10 min, after deparaffinization in a microwave oven. The sections were then dipped into PBS containing 3% H_2_O_2_ and 0.1% Triton X-100 to quench endogenous peroxidase activity. After treatment with 10% normal donkey serum (Jackson ImmunoResearch Labs Inc., West Grove, PA, USA) to block non-specific binding signals, the slides were incubated with NR1D2-specific antibody (Proteintech Group, Inc.) overnight at 4 °C and then incubated with a mouse biotinylated secondary antibody (Abcam, Cambridge, MA, USA) for 2 h at room temperature. Immunoreactivity with NR1D2 was visualized using streptavidin-peroxidase and 3,3′-diaminobenzidine (Maixin Bio, Fuzhou, China).

### RNA extraction and real-time PCR

RNA was extracted from cells and subjected to real-time PCR. Briefly, RNA was extracted following a standard TRIzol protocol, and real-time PCR was performed with the ABI Step One System (Applied Biosystems, Foster City, CA, USA) using the SYBR Premix Ex Taq II kit (TaKaRa Bio, Inc.). To detect the relative expression of NR1D2 mRNA and mature miR-210, their expression levels were normalized to β-actin and U6 snRNA, respectively. The qRT-PCR primers are listed in Supplementary Table 1.

### ELISA

The IL-6 concentration was measured with a RayBio Human IL-6 ELISA Kit (RayBio Inc.).

### Statistical analysis

All experiments in this study were performed independently at least three times. Data are shown as the means plus standard errors of the mean (SEMs). A P-value < 0.05 was considered significant.

## Results

### MicroRNA-210 is upregulated in NOA patients and expressed in early testicular germ cells, with particularly high expression in testes with cryptorchidism

**Figure 1:**
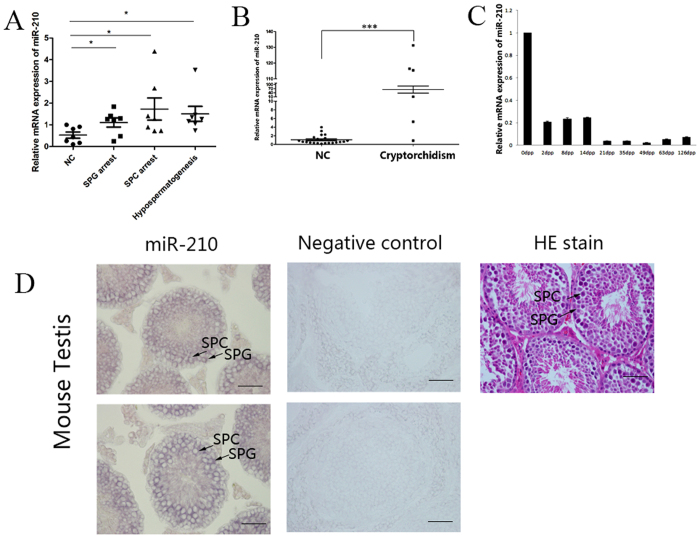
MicroRNA-210 is upregulated in NOA patients and expressed in early testicular germ cells, with particularly high expression in testes with cryptorchidism. (**A**) The expression of miR-210 in the testes of normal controls (OA) and patients with MA (SPG arrest, SPC arrest or hypospermatogenesis) was examined by real-time PCR. (**B**) The expression of miR-210 in the testes of normal controls (OA) and patients with cryptorchidism, as determined by real-time PCR. (**C**) The expression of miR-210 in the testes of mice at various experimental time points, as determined by real-time PCR. (**D**) Localization of miR-210 in the testes of adult mice by LNA-based miRNA *in situ* hybridization. Representative haematoxylin and eosin staining; hybridization signals (purple) for miR-210 and negative controls are shown. NC, normal controls; SPG, spermatogonia; SPC, spermatocyte. Scale bar = 50 μm. All data are presented as the means ± SEMs from at least three independent experiments. *p < 0.05, ***p < 0.001.

Our previous microRNA array analyses have shown that miR-210 expression is upregulated in the testes of infertile men with MA^13^. To confirm this finding, we used real-time PCR to detect miR-210 expression in 7 normal controls, 7 SPG arrest samples, 6 SPC samples and 7 hypospermatogenesis samples. We observed a significant upregulation of miR-210 expression in testes obtained from all NOA patients compared with that in normal controls, using real-time PCR (Fig. 1A). In addition, in mouse seminiferous tubules that displayed normal spermatogenesis, miR-210 expression was readily detectable in the spermatogonia and spermatocytes (Fig. 1D). In agreement with the microRNA microarray results, miR-210 was significantly upregulated in testicular specimens displaying MA, such as spermatogonia arrest and spermatocyte arrest. This upregulation may not be exclusive to these patients because miR-210 expression was altered in infertile patients with hypospermatogenesis.

Moreover, miR-210 was highly upregulated in patients with cryptorchidism (Fig. 1B). Real-time PCR analysis revealed that miR-210 expression increased significantly in 6 testes with cryptorchidism compared with that of 22 normal controls. Simultaneously, miR-210 expression decreased from the testes of new-born mice to those of adult mice. In testes from new-born mice, miR-210 expression was ten times greater than that in testes from adult mice (Fig. 1C). These data suggest that miR-210 is involved in spermatogenesis or the development of cryptorchidism.

### NR1D2 is located in spermatogonia and declines in patients with cryptorchidism

**Figure 2:**
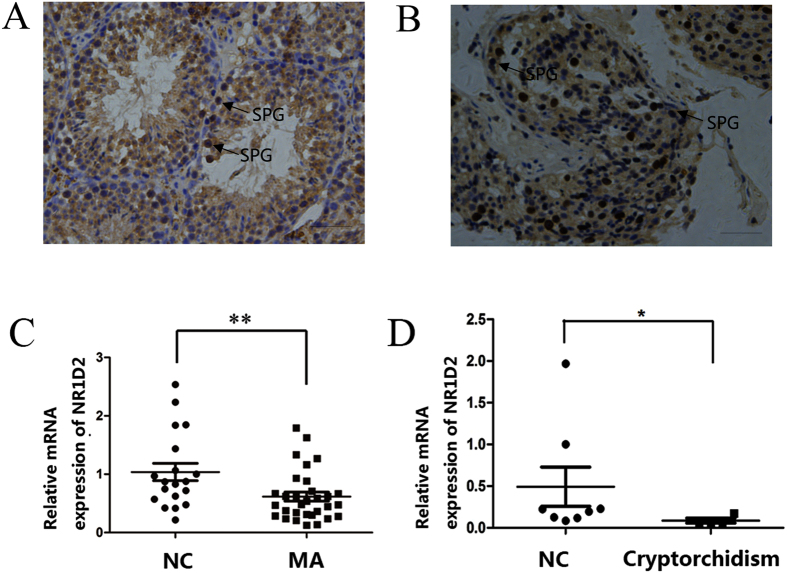
NR1D2 is located in spermatogonia and declines in cryptorchidism. (**A**) Immunohistochemical analysis of the NR1D2 protein in mouse testicular tissue. (**B**) Immunohistochemical analysis of the NR1D2 protein in human testicular tissue from patients with maturation arrest. SPG, spermatogonia; scale bar = 50 μm for all images. (**C**) NR1D2 mRNA levels in the testes of normal controls (OA) and patients with maturation arrest, as detected by real-time PCR. (**D**) NR1D2 mRNA levels in the testes of normal controls (OA) and patients with cryptorchidism, as detected by real-time PCR. *p < 0.05, **p < 0.01.

NR1D2 encodes a member of the nuclear hormone receptor family, specifically the NR1 subfamily of receptors. The encoded protein functions as a transcriptional repressor and plays a role in circadian rhythms and carbohydrate and lipid metabolism^19^,^20^. Our previous studies found changes in the expression of immune-related genes in patients with cryptorchidism, including the interleukin family and related nuclear receptor family (Figure S2). NR1D2 has not been reported to exhibit any relationship with reproduction. Therefore, the location and expression of NR1D2 in the testis are still unknown. Thus, we explored whether NR1D2 is expressed in spermatogonia or spermatocytes in the testis. Immunohistochemical analyses showed that NR1D2 was in the spermatogonia in the mouse testis (Fig. 2A) and in patients with MA (Fig. 2B), consistent with the localization of miR-210 in human testicular tissues. Additionally, real-time PCR showed that NR1D2 expression was lower in 30 MA patient samples than in 19 normal controls (Fig. 2C). Moreover, NR1D2 expression was significantly downregulated in 7 cryptorchidism samples relative to 8 normal controls (Fig. 2D).

### NR1D2 is directly targeted by miR-210

**Figure 3:**
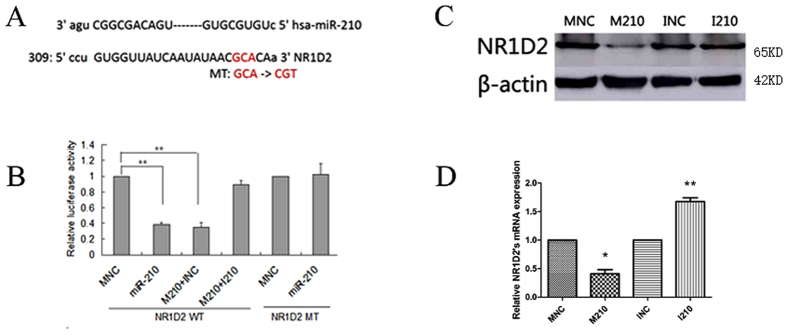
NR1D2 is directly targeted by miR-210. (**A**) Putative binding sites for human miR-210 were predicted in the 3′-UTR of NR1D2 mRNA. The mutated bases of predicted miR-210 binding sites are underlined, namely MT-NR1D2 3′-UTR. (**B**) miR-210 targeted the 3′-UTR of NR1D2. Luciferase reporters containing either miR-210 putative binding sites from the wild-type NR1D2 3′-UTR (WT NR1D2-3′-UTR) or mutated NR1D2 3′-UTR (MT NR1D2-3′-UTR) were co-transfected with the indicated microRNA mimics into 293T cells. Luciferase activity was measured 30 h after transfection. (**C**) The NR1D2 protein was quantified by Western blotting. NT-2 cells were harvested 48 h after transfection with RIPA lysis buffer. Gels were run under the same experimental conditions (120 V for 90 mins). (**D**) NR1D2 mRNA expression was evaluated by real-time PCR. Total RNA was extracted 48 h after transfection using a standard TRIzol protocol. WT: wild-type, MT: mutant, MNC: normal control mimics, M210: miR-210 mimics, INC: normal control inhibitors, I210: miR-210 inhibitors. All data are presented as the means ± SEMs from at least three independent experiments. *p < 0.05, **p < 0.01.

To investigate the function of miR-210 in cryptorchidism, we used the microRNA target prediction algorithm miRanda to predict potential targets of miR-210. This analysis revealed that the 3′-UTR of NR1D2 mRNA contains one presumptive miR-210 binding site (positions 309–315) (Fig. 3A). As described above, NR1D2 was upregulated in patients with cryptorchidism. NR1D2 is a transcriptional factor that participates in circadian rhythms, inflammation, carbohydrate and lipid metabolism^19^,^21^,^22^. To determine whether NR1D2 is an authentic target of miR-210, miR-210 mimics/negative control or inhibitor of miR-210/inhibitor control were transfected into NT-2 cells. There was a significant reduction in NR1D2 mRNA levels in miR-210-transfected NT-2 cells (Fig. 3D). In addition, miR-210 downregulated the NR1D2 protein level in NT-2 cells (Fig. 3C). These data indicate that miR-210 modulates NR1D2 expression at the transcriptional and translational levels. To determine whether NR1D2 is a direct target of miR-210, we constructed Renilla luciferase reporters containing either the wild-type full-length NR1D2 3′-UTR or the mutant form of its seeding sites. We analysed 293T cells transfected with constructs carrying the luciferase gene fused with the 3′-UTR of NR1D2 or cells co-transfected with the parent luciferase expression vector and miR-210 mimics or negative control mimics. This resulted in an approximately 37% reduction in luciferase activity, whereas the inhibition of miR-210 expression led to the recovery of luciferase activity. Mutation of the predicted binding site of miR-210 within the 3′-UTR of NR1D2 (MT-NR1D2-3′-UTR) abolished the silencing effect of miR-210 on luciferase activity (Fig. 3B).

The results above demonstrate that miR-210 negatively modulates the expression of NR1D2 by directly binding to the seed sequence of the NR1D2 3′-UTR.

### miR-210 regulates IL-6 by targeting NR1D2

**Figure 4:**
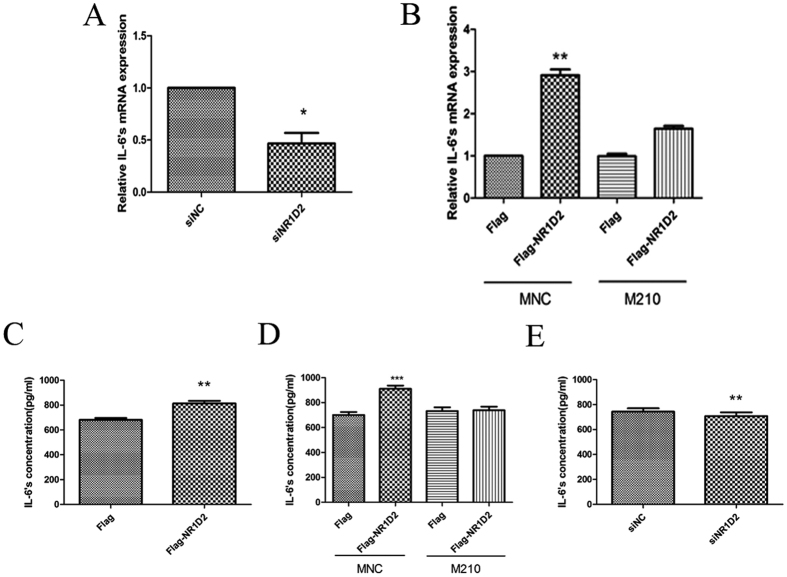
miR-210 regulates IL-6 by targeting NR1D2. (**A**,**E**) Silencing of NR1D2 resulted in downregulated IL-6 mRNA and secretion levels. NR1D2 siRNA (si.NR1D2) or negative control (si.NC) were transfected into NT-2 cells at 200 nM and harvested 48 h after transfection. (**C**) Upregulated IL-6 secretion levels in association with NR1D2 overexpression. (**B**,**D**) miR-210 reversed NR1D2-induced IL-6 mRNA expression and secretion. Flag-NR1D2 and miR-210 mimics were co-transfected into NT-2 cells. Cells were harvested 48 h after transfection. MNC: normal control mimics, M210: miR-210 mimics. All data are presented as the means ± SEMs from at least three independent experiments. *p < 0.05, **p < 0.01, ***p < 0.001.

NR1D2 plays a role in circadian rhythms, inflammation, and carbohydrate and lipid metabolism^19^,^21^,^22^. Additionally, the encoded protein functions as a transcriptional repressor^23^. Because NR1D2 may be involved in the expression of inflammatory factors, we investigated whether cytokines are regulated by NR1D2. Among the numerous inflammatory cytokines, interleukin-6 (IL-6) showed NR1D2-specific regulation. To verify whether NR1D2 is involved in IL-6 regulation, we specifically silenced NR1D2 (si.NR1D2) in NT-2 cells by RNA interference (RNAi). Transfection of NT-2 cells with si.NR1D2 caused a significant decrease in IL-6 at both the transcription (Fig. 4A) and secretion (Fig. 4E) levels. The over-expression of NR1D2 by fusion of the CDS and 3′-UTR (740 bp adjacent to CDS, including the seed sequence) region of NR1D2 with the vector p3XFLAG in NT-2 cells increased transcription and secretion (Fig. 4B,C). In contrast, IL-6 expression was significantly downregulated in NT-2 cells co-transfected with miR-210 and p3XFLAG-NR1D2 compared with in NT-2 cells separately transfected with p3XFLAG-NR1D2 (Fig. 4B,D).

### miR-210 expression increases in the mouse model of cryptorchidism

**Figure 5:**
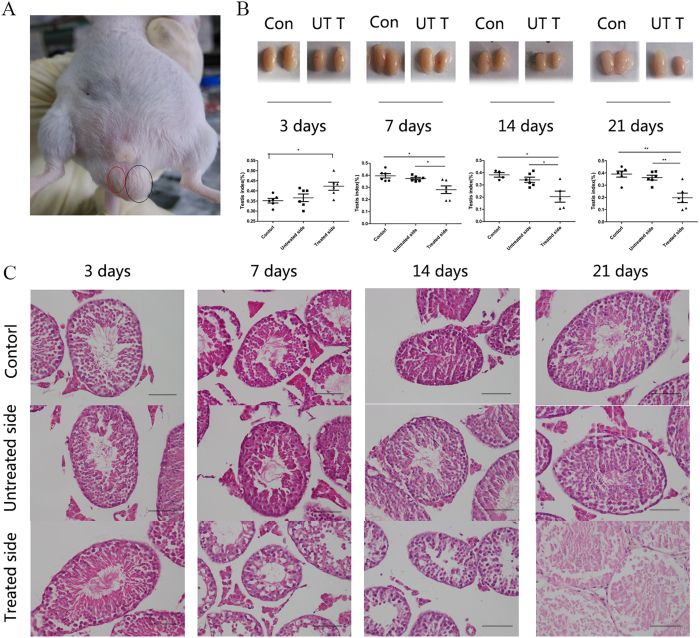
The mouse cryptorchidism model. (**A**) The normal (black circle) and cryptorchid (red circle) mouse testes. (**B**) Photographs of the testes of mice with cryptorchidism (the top panel) and the corresponding testis index (the bottom panel). The testis samples were obtained at 3, 7, 14, 21 days after the cryptorchidism surgery on mice. The testis index was expressed as (testis weight/body weight) *100%. Con: control, UT: Untreated side, T: Treated side. (**C**) HE analysis of the testes of mice with cryptorchidism. The testis sections were obtained at 3, 7, 14, 21 days after the cryptorchidism surgery on mice. The testes from the treated and untreated sides 3 days after surgery showed no differences from those of control mice. By 7 days after surgery, the testes with cryptorchidism showed internal morphological damage and an absence of mature sperm. By 14 days after surgery, cavitation was observed in the testes with cryptorchidism in addition to a damaged morphology and lack of mature sperm. By 21 days after surgery, the morphology of sperm from the testes with cryptorchidism was completely destroyed, with no internal structure remaining in all levels of germ cells and mature sperm. At the same time, spermatogenesis did not change significantly in the untreated testes compared to the control mice. Bar = 50 μm for all images. *p < 0.05.

To determine the expression of miR-210 at different stages of cryptorchidism, we established a mouse model of cryptorchidism. Mice were divided into four groups of 12 mice each. In each group, only 6 mice were treated with surgery; the remaining 6 mice were kept as controls. When mice were under anaesthesia, the testis on one side was fixed to the abdominal cavity using the fat pad near the testis for suturing (Fig. 5A). After surgery, the 4 groups of mice were kept in an SPF-level animal facility for 3, 7, 14 or 21 days. After the corresponding number of days, mouse testis samples were obtained, and the image contrast and testicular weight were analysed. The testes with cryptorchidism decreased significantly in size and weight; by the third day, they increased slightly in size and weight, possibly due to oedema (Fig. 5B). At each time point, the testis contralateral to the operation side did not change significantly in size or weight (Fig. 5B). Correspondingly, the testis index (ratio = (testis weight/body weight) *100%) of the testes with cryptorchidism was downregulated compared with that of controls and untreated testes at 7, 14 and 21 days after surgery.

Mutations in the genes insulin-like 3 (Insl3) and relaxin/insulin-like family peptide receptor 2 (also known as LGR8) are associated with cryptorchidism in humans, and changes in their expression have been observed in this model^24^ (Figure S1). A morphological comparison of the mature sperm from HE-stained testes from the affected and contralateral sides 3 days after surgery showed no differences from those of normal mice (Fig. 5C). By 7 days after surgery, the testes with cryptorchidism showed internal morphological damage and an absence of mature sperm (Fig. 5C). By 14 days after surgery, cavitation was observed in the testes with cryptorchidism in addition to a damaged morphology and lack of mature sperm (Fig. 5C). By 21 days after surgery, the morphology of sperm from the testes with cryptorchidism was completely destroyed, with no internal structure remaining in all levels of germ cells and mature sperm. At the same time, spermatogenesis did not change significantly in the untreated testes compared to the control mice (Fig. 5C). Both the appearance and internal structure of cryptorchidic testes were destroyed; therefore, the testes could not generate mature sperm, which led to infertility.

**Figure 6:**
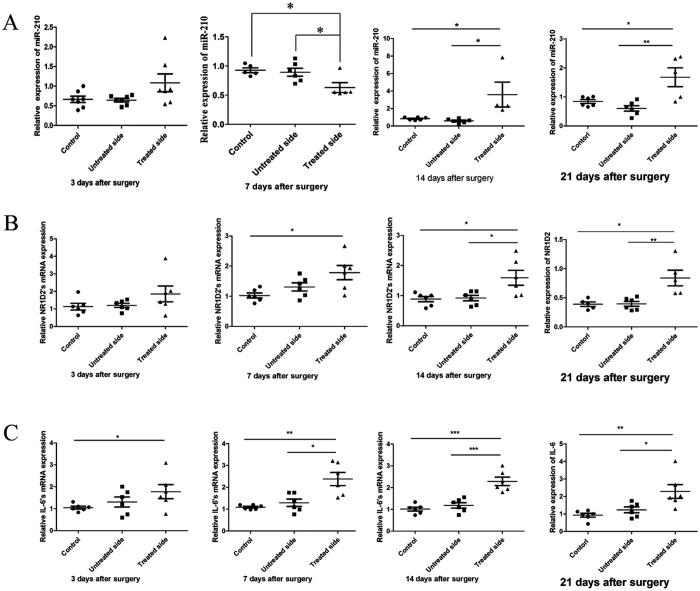
miR-210, NR1D2 and IL-6 expression in mice with cryptorchidism. (**A**) miR-210 expression in mice with cryptorchidism. (**B**) NR1D2 expression in mice with cryptorchidism. (**C**) IL-6 expression in mice with cryptorchidism. Tissue RNA was extracted with TRIzol and examined by real-time PCR. All data are presented as the means ± SEMs from at least three independent experiments. *p < 0.05, **p < 0.01, ***p < 0.001.

Next, the mRNA expression levels of the relevant molecules were analysed. Except for a slight decline 7 days after surgery, miR-210 expression increased significantly at 14 and 21 days after surgery (Fig. 6A). These results are consistent with the analysis of testis tissue from patients with cryptorchidism. Real-time PCR analysis showed an increase in the expression of NR1D2 and IL-6 (Fig. 6B,C), which was not exactly consistent with the results of the *in vitro* experiments.

## Discussion

The absence of one or both testes in the scrotum, defined as cryptorchidism, is a very common birth defect of the male genitourinary system. In special cases, cryptorchidism can develop as late as during young adulthood. Approximately 3% of full-term and 30% of premature infant boys are born with at least one undescended testis^8^. However, approximately 80% of testes with cryptorchidism have descended by the first year of life (the majority within three months), resulting in an overall incidence of cryptorchidism of 1%. Cryptorchidism is distinct from monorchism, the condition of having only one testicle. In recent decades, several studies have focused on microRNA functions during spermatogenesis, but only a few microRNA studies have concentrated on idiopathic diseases of the genitourinary system, including cryptorchidism. In this study, we observed aberrant increases in miR-210 in nonobstructive azoospermia patients and cryptorchidic patients. However, miR-210 is located in the spermatogonia and spermatocytes in human testicular tissues. This pattern suggests that the aberrant increase in miR-210 in the early stage of spermatogenesis is associated with azoospermia and cryptorchidism.

Furthermore, NR1D2 was aberrantly expressed in patients with MA or cryptorchidism in this study. NR1D2, also called Rev-erbβ, is very similar to NR1D1 (nuclear receptor family 1 member 1). The major known function of NR1D2, together with NR1D1, is regulating circadian rhythm and metabolism^25^. NR1D1 and NR1D2 cooperate in regulating core clock function and mediating the interplay between the circadian rhythm and metabolism^26^. NR1D1 and NR1D2 repress the positive (Bmal1) and negative (Cry1 and NR1D1) limbs of the core clock^27^. Additionally, NR1D1 and NR1D2 mediate the interaction between the cellular clock and metabolism with BMAL1 and CLOCK/NPAS2 28. However, few reports address the expression of NR1D2 and its function in reproduction. Herein, we report that NR1D2 was localized in spermatogonia and spermatocytes and was downregulated in MA patients and those with cryptorchidism. This result reveals that NR1D2 plays a role in spermatogenesis and affects the occurrence of cryptorchidism.

To validate whether these abnormal changes are connected, we performed a series of experiments and demonstrated that miR-210 negatively modulates NR1D2 expression by directly binding to the seed sequence of the NR1D2 3′-UTR. Thus, the aberrant downregulation of NR1D2 in patients with MA and cryptorchidism may be caused by the aberrant increase in miR-210.

Furthermore, changes in immune parameters with the disruption of circadian rhythms have been linked to inflammatory pathologies^19^. Thus, there is compelling evidence that NR1D1 affects immunity. NR1D1 may negatively regulate an unidentified inhibitor(s) that blocks NF- κB activation and thereby induce IL-6 expression^29^,^30^. Because the NR1D2 protein in mice is ~96% identical to the NR1D1 protein, NR1D2 may be associated with IL-6 expression. Real-time PCR and ELISA experiments were performed to verify that NR1D2 can promote IL-6 expression and secretion as proposed. Additionally, miR-210 reverses these changes in IL-6 by inhibiting NR1D2 expression.

To confirm the mechanism that underlies the role of miR-210 in cryptorchidism, we mimicked the effect of cryptorchidism using a surgical procedure in mice. miR-210 expression increased significantly in this cryptorchidism model. However, there was a concomitant increase in NR1D2 expression. Further analysis is required to confirm whether the observed NR1D2 upregulation resulted from the inflammatory response in the model system. In this model, many genes related to inflammation changed significantly (Figure S5). Cryptorchidism caused a stress response and inflammatory reaction. Nevertheless, significant increases in miR-210 expression were demonstrated in tissues derived from both patients with cryptorchidism and mouse testes subjected to experimental cryptorchidism. In summary, a significant increase in miR-210 was observed in the testes of NOA patients, particularly in cryptorchidism. We demonstrated that miR-210 acts as an upstream regulator of NR1D2 function in human cryptorchidism. The mouse model of cryptorchidism verified that miR-210 expression was correlated with cryptorchidism.

Our current preliminary research partially revealed the function of NR1D2 and established the regulatory relationship between miR-210 and NR1D2. Even more importantly, this study is the first to confirm the relationship between miR-210 and cryptorchidism. miR-210 may serve as a biological marker of cryptorchidism in clinical tests and may be used to predict the occurrence of cryptorchidism.

## Additional Information

**How to cite this article**: Duan, Z. *et al*. The functional and predictive roles of miR-210 in cryptorchidism. *Sci. Rep.* **6**, 32265; doi: 10.1038/srep32265 (2016).

## Supplementary Material

Supplementary Information
