# Supplementary material for: The functional and predictive roles of miR-210 in cryptorchidism
Source: Sci Rep. 2016 Aug 26;6:32265. doi: 10.1038/srep32265 (PMC5000482; doi:10.1038/srep32265)
Supplement: Supplementary Information [file srep32265-s1.pdf]

Title: The functional and predictive roles of miR-210 in cryptorchidism

Zhengzheng Duan[1], Helong Huang[1], Fei Sun[1,2]*

Supplementary Information

Fig. S1.Insl3 and Lgr8's expression in cryptorchidism mice.

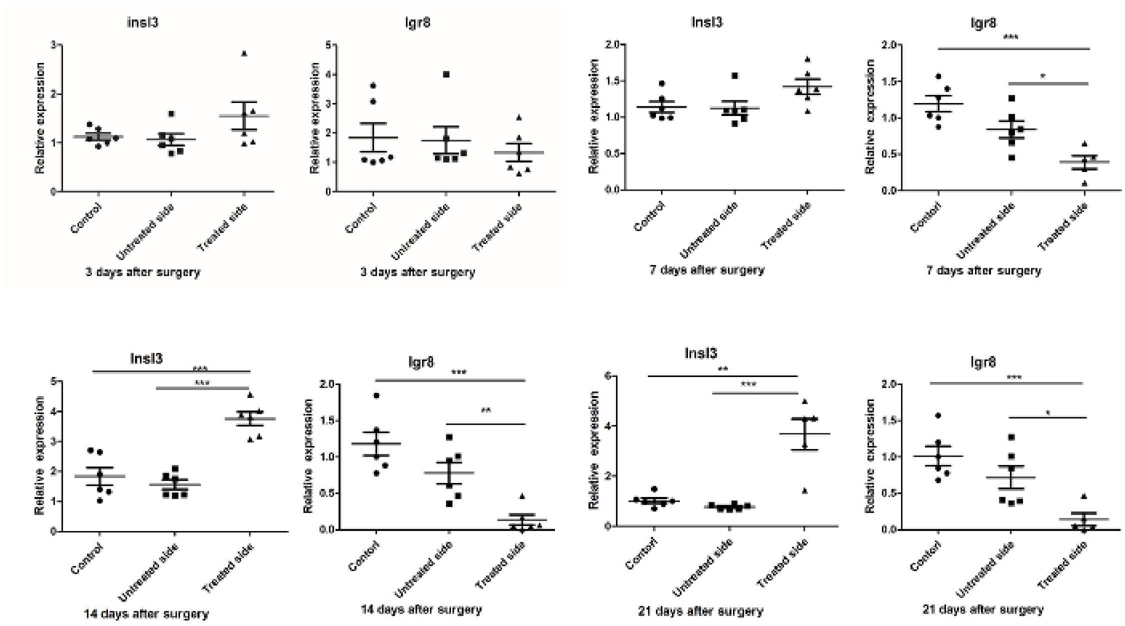

Figure.S2. Different genes' expression in cryptorchidism patients and normal controls.

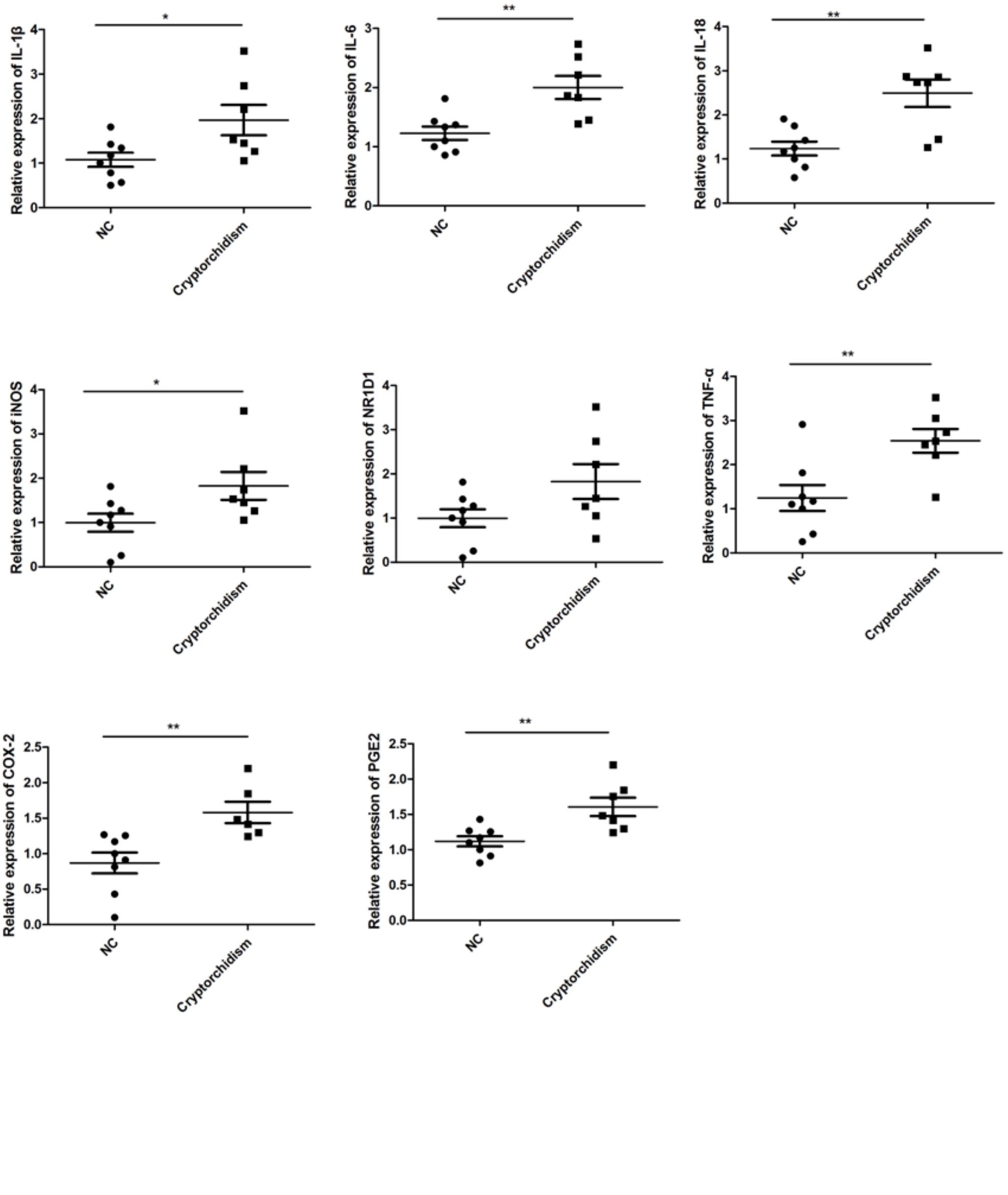

Figure.S3. The original images of Western Blot.

The upper blots of the two images are β-actin and NR1D2, meanwhile the other blots in the images were irrelevant to this article.

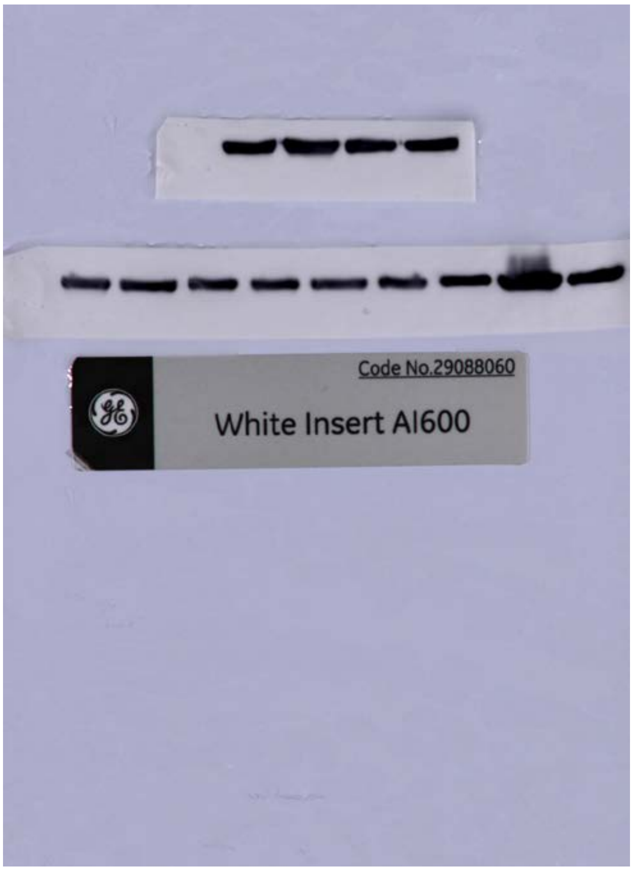

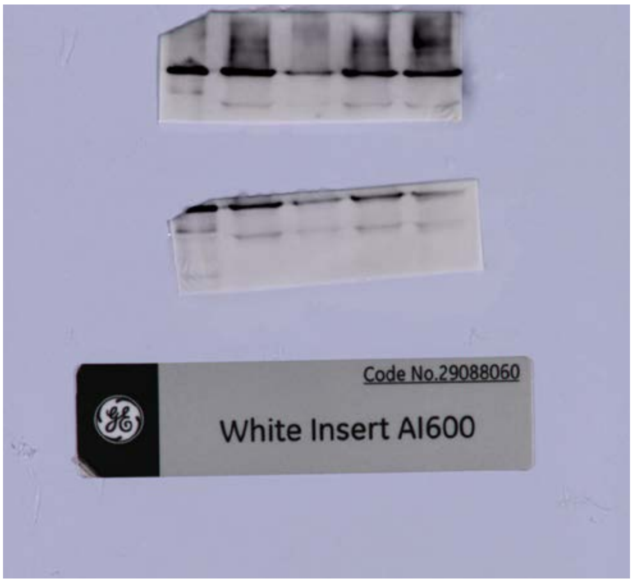

Figure.S4. MiR-210's expression in different cell lines.

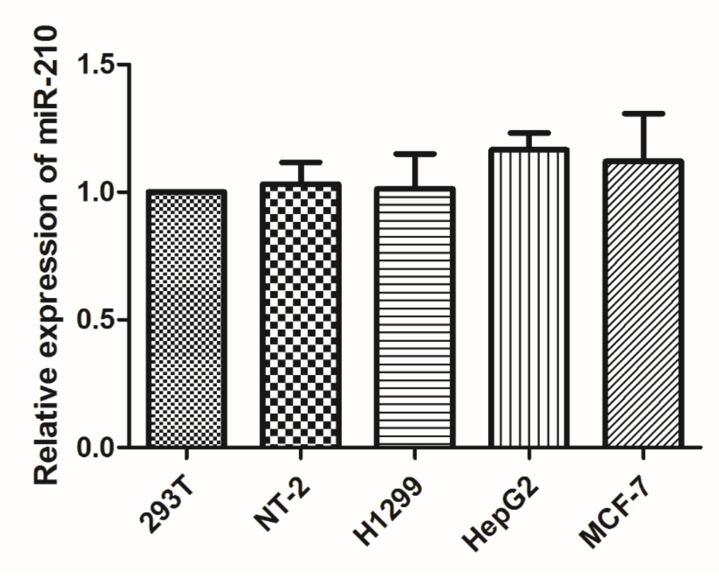

Figure.S5. Different genes' expression in cryptorchidism mice.

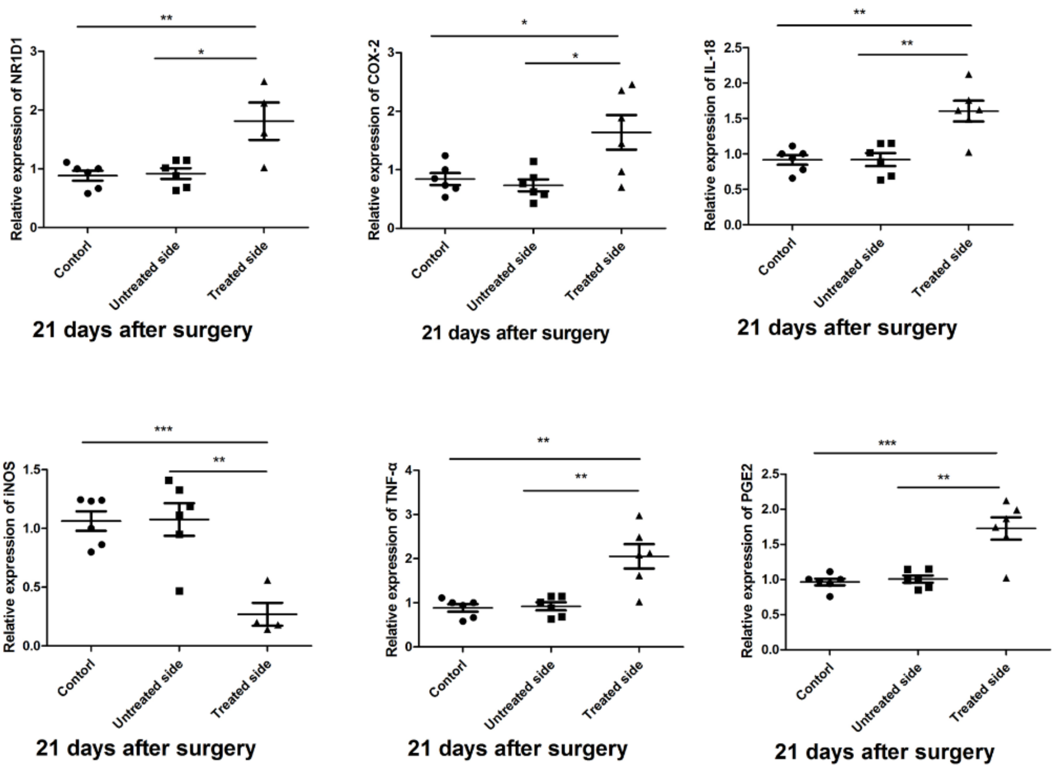

Table.1.	Real-time PCR Primers	
	Forward primer	Reverse primer
human β-actin	5'-TGGCACCCAGCACAATGAA-3'	5'-CTAAGTCATAGTCCGCCTAGAAGCA-3'
mouse β-actin	5'-TGGCTCCTAGCACCATGAA-3'	5'-CTCAGTAACAGTCCGCCTAGAAGCA-3'
human NR1D2	5'-TGGTGCGCTTTGCTTCGT-3'	5'-TGCGGCTTAGGAACATCAC-3'
mouse NR1D2	5'-AGCCCAAAGATGAGCCTAATTC-3'	5'-GGTTCTCTATCTGGCTGATGTCA-3'
human IL-6	5'-GGTGCCCATGCTACATTTGC-3'	5'-GGTTCAGGTGACACCACAAATTC-3'
mouse IL-6	5'-CACGGCCTTCCCTACTTCAC-3'	5'-ACAGGTCTGTTGGGAGTGGTA-3'
mouse Insl3	5'-GGTGCCCATGCTACATTTGC-3'	5'-GCCTGTGGTCCTTGCTTACTG-3'
mouse Lgr8	5'-GCCTGTGGTCCTTGCTTACTG-3'	5'-CCTGGAGATCCACCCTGTATCAG-3'
human NR1D1	5'-TGGTGCGCTTTGCTTCGT-3'	5'-TGCGGCTTAGGAACATCAC-3'
mouse NR1D1	5'-GCTTCTCCGTTGGCATGTCT-3'	5'-CCAAGTTCATGGCGCTCT-3'
human iNOS	5'-GTGGCCCACACCCTGAAG-3'	5'-CCTCGACCTGCTCCTCATTC-3'
mouse iNOS	5'-TGCTCCCTTCCGAAGTTTCTG-3'	5'-TCATGCGGCCTCCTTTGAG-3'
human p21	5'-CGCTAATGGCGGGCTG-3'	5'-CGGTGACAAAGTCGAAGTTCC-3'
mouse p21	5'-GTGATTGCGATGCGCTCATG-3'	5'-TCTCTTGCAGAAGACAATC-3'
human COX-2	5'-GCTAGCCCAAGAATATTGTC-3'	5'-GTGGCTGAACAAATTAACGAAGCAT-3'
mouse COX-2	5'-GGGTGTCCCTTCACTTCTTTCA-3'	5'-GGGAGGCACTTGCATTGATG-3'
human TNF-α	5'-GAGGCCAAGCCCTGCG-3'	5'-CGGGCCGATTGATCTCAGC-3'
mouse TNF-α	5'-CTGTCTACTGAACTTCGGGGT-3'	5'-GAGTGTGAGGGTCTGGGC-3'
human PGE-2	5'-AGACGGACCACCTCATTCTC-3'	5'-AGGCCTAAGGATGGCAAAGA-3'
mouse PGE-2	5'-AGGTTAATGCGCTCAGTCCT-3'	5'-CAGCCCCTTACACTTCTCCA-3'
human IL-18	5'-GGAAATCGGCCTCTATTTGAAGA-3'	5'-GTCCGGGGTGCATTATCTCT-3'
mouse IL-18	5'-ACTTTGGCCGACTTCACTGT-3'	5'-GGGTTCACTGGCACTTTGAT-3'
human IL-1β	5'-GACCTGAGCACCTTCTTTCCCTTC-3'	5'-GCAGTTCAGTGATCGTACAGGTGC-3'
mouse IL-1β	5'-TGGGCCTCAAAGGAAAGAAT-3'	5'-CAGGCTTGTGCTGCTTGT-3'

**Table.2**	
**Name**	**Sequence (5' to 3')**
**LNA scramble-miR**	GTGTAACACGTCTATACGCCCA
**LNA hsa-miR-210**	TCAGCCGCTGTCACACGCACAG
**Inhibitor NC**	CAGUACUUUUGUGUAGUACAA
**miR-210 inhibitor**	UCAGCCGCUGUCACACGCACAG
**mimic NC & si.NC**	UUCUCCGAACGUGUCACGUTT
	ACGUGACACGUUCGGAGAATT
**miR-210 mimic**	CUGUGCGUGUGACAGCGGCUGA
	AGCCGCUGUCACACGCACAGUU
